# Heart Disease Characterization and Myocardial Strain Analysis in Patients with *PACS1* Neurodevelopmental Disorder

**DOI:** 10.3390/jcm12124052

**Published:** 2023-06-14

**Authors:** Ana Latorre-Pellicer, Laura Trujillano, Julia del Rincón, Mónica Peña-Marco, Marta Gil-Salvador, Cristina Lucia-Campos, María Arnedo, Beatriz Puisac, Feliciano J. Ramos, Ariadna Ayerza-Casas, Juan Pié

**Affiliations:** 1 Unit of Clinical Genetics and Functional Genomics, Department of Pharmacology-Physiology, School of Medicine, Universidad de Zaragoza, CIBERER-GCV02 and IIS-Aragon, E-50009 Zaragoza, Spain; alatorre@unizar.es (A.L.-P.);; 2 Department of Clinical and Molecular Genetics Hospital Vall d’Hebron, E-08035 Barcelona, Spain; 3 Unit of Clinical Genetics, Department of Paediatrics, Service of Paediatrics, Hospital Clínico Universitario Lozano Blesa, School of Medicine, Universidad de Zaragoza, CIBERER-GCV02 and IIS-Aragon, E-50009 Zaragoza, Spain; 4 Unit of Paediatric Cardiology, Service of Paediatrics, Hospital Universitario Miguel Servet, E-50009 Zaragoza, Spain

**Keywords:** Schuurs-Hoeijmakers syndrome, *PACS1* neurodevelopmental disorder, *PACS1*-NDD, congenital heart disease, myocardial strain analysis

## Abstract

Background: *PACS1* neurodevelopmental disorder (*PACS1*-NDD) (MIM# 615009) is a rare autosomal dominant disease characterized by neurodevelopmental delay, dysmorphic facial features, and congenital malformations. Heart disease (HD) is frequently present in individuals with *PACS1*-NDD, but a compressive review of these anomalies and an evaluation of cardiac function in a cohort of patients are lacking. Methods: (i) Cardiac evaluation in 11 *PACS1*-NDD patients was conducted using conventional echocardiography. (ii) Heart function was assessed by tissue Doppler imaging, and two-dimensional speckle tracking was performed in seven patients and matched controls. (iii) This systematic review focused on determining HD prevalence in individuals with *PACS1*-NDD. Results: In our cohort, 7 of 11 patients presented HD. (Among them, three cases of ascending aortic dilatation (AAD) were detected and one mitral valve prolapse (MVP).) None of the patients showed echocardiographic pathological values, and the left global longitudinal strain was not significantly different between patients and controls (patients −24.26 ± 5.89% vs. controls −20.19 ± 1.75%, *p* = 0.3176). In the literature review, almost 42% (42/100) of individuals with *PACS1*-NDD reportedly experienced HD. Septal defects were the most common malformation, followed by patent ductus arteriosus. Conclusions: Our results show a high prevalence of HD in *PACS1*-NDD patients; in this way, AAD and MVP are reported for the first time in this syndrome. Furthermore, a detailed cardiac function evaluation in our cohort did not reveal evidence of cardiac dysfunction in individuals with *PACS1*-NDD. Cardiology evaluation should be included for all individuals with Schuurs-Hoeijmakers syndrome.

## 1. Introduction

Schuurs-Hoeijmakers syndrome (SHMS), or *PACS1* neurodevelopmental disorder (*PACS1*-NDD) (MIM# 615009), is a recently described rare autosomal dominant disease associated with developmental delay and intellectual disability [1,2]. *PACS1*-NDD is caused by a recurrent genetic variant in the gene *PACS1* (NM_018026.3; c.607C>T, p.(Arg203Trp)), which encodes the phosphofurin acid cluster sorting 1 (PACS-1) protein [1].

Since 2012, approximately 100 patients with *PACS1*-NDD have been reported in the literature [2,3,4]. This syndrome has a recognizable craniofacial gestalt that includes downslanting palpebral fissures, ocular hypertelorism, full and arched eyebrows, long eyelashes, low-set ears, bulbous nasal tip, anteverted nares, broad nasal bridge, thin upper lip, and downturned corners of the mouth, among other signs [2,4,5]. Initially, similarities between the facial features of *PACS1*-NDD and Cornelia de Lange syndrome (CdLS) were identified, and a clinical diagnosis of CdLS was even suggested for the first two patients reported to have *PACS1*-NDD [1]. However, as more patients were described, it became apparent that the facial features of *PACS1*-NDD corresponded to a specific entity, with strong similarities to *PACS2* (MIM# 618067)- and *WDR37* (MIM#618652)-related syndromes [6,7].

All patients described in the literature have neurodevelopmental delay with intellectual disability and psychomotor retardation [2,3,4]. In most of the patients, walking is achieved before the age of 4. However, language skills are usually more affected, with delays ranging from mild to severe, and some patients do not develop verbal communication [2]. Seizures are a frequent clinical complication that can be well controlled by antiepileptic drugs [8], and about 30% of *PACS1*-NDD patients show behavior within the autism spectrum [4,9].

The earlier descriptions of *PACS1*-NDD patients also revealed a wide range of congenital anomalies, including congenital heart diseases (CHDs), brain abnormalities, and ophthalmologic defects. Coloboma is the main ophthalmological manifestation [9], although other abnormalities, such as myopia, strabismus, nystagmus, microcornea, and microphthalmia, have been described [10]. Brain defects may also be present, with hypoplasia and partial agenesis of the cerebellar vermis being the most common findings [9]. Finally, congenital heart anomalies have been reported in about 40% of individuals with *PACS1*-NDD, but little is known about the clinical implications of these anomalies and their effects on the quality of life of patients [9].

The aim of this study was to comprehensively review the heart diseases found in *PACS1*-NDD patients and to evaluate cardiac function in a well-defined cohort of patients using conventional echocardiography and the two-dimensional speckle-tracking technique to assess the clinical implications of cardiac pathology in *PACS1*-NDD.

## 2. Materials and Methods

Patient cohort: This study included 11 patients (9 unrelated individuals and 2 twin siblings) with *PACS1*-NDD (6 males, 5 females, aged 2–35 years). Cardiac function evaluation by echocardiography was performed in seven of them and seven healthy controls of the same age and sex. All patients, or their parents or guardians, signed a written consent form to participate in this study, which was approved by the Ethics Committee of Clinical Research from the Government of Aragón (Spain) (CEICA; PI16/225). Clinical data were collected using a standard restricted-term questionnaire, and detailed phenotypes of the individuals were entered by the patients’ clinician using the Human Phenotype Ontology (HPO) nomenclature.

Genetic testing: The genetic variant in the gene *PACS1* (NM_018026.3; c.607C>T, p.(Arg203Trp)) was confirmed in all the patients by Sanger sequencing. Genomic DNA was isolated from blood lymphocytes using the conventional phenol–chloroform–isoamyl alcohol method or from a buccal swab using prepIT.L2P (DNA Genotek Inc. Ottawa, ON, Canada, PT-L2P-5), according to the manufacture’s protocols. Quality and concentration of gDNA were determined using both the Qubit Fluorometric Quantitation (Thermo Fisher Scientific, Inc., Waltham, MA, USA) and Nanodrop 2000 (Thermo Fisher Scientific, Inc., Waltham, MA, USA). PCR amplifications were performed using the primers F: 5′CTCCAGAACCCCTCAAGGAC3′ and R: 5′CTGTGACTCAAAGGCCAACA3′. PCR products were sequenced on the ABI3730xl Capillary Electrophoresis Sequencing System (Applied Biosystems, Inc., Waltham, MA, USA), according to the manufacturer’s protocol. Sequences were analyzed and compared to the reference sequences using the Analysis Module Variant Analysis (VA) software version 2.1.3 (Applied Biosystem, Inc., Waltham, MA, USA) and Ensembl and NCBI databases.

Anthropometric measurements and clinical records: Individuals with *PACS1*-NDD underwent anthropometric and physical examinations, and medical records were collected from their clinical histories. Weight was measured in kilograms using an AMGI-IMSA model, and height was measured in centimeters using the Harpenden model tallimeter. Weight and height percentiles and their distance from the mean in standard deviations (z-scores) were calculated in patients between 2 and 17 years of age using the Endocrinoped application (Spanish reference charts 2010) (http://www.webpediatrica.com/endocrinoped/antropometria.php (accessed on 1 March 2023)). Heart rate, systolic blood pressure, and diastolic blood pressure were monitored in some patients. In individuals under 18 years of age, blood pressure percentiles were calculated according to age and height using the MSD manual calculator (https://www.msdmanuals.com/es-es/professional/multimedia/clinical-calculator/percentiles-de-tensión-arterial-para-niños-2-17-años (accessed on 1 September 2022)). In adult patients, blood pressure values less than 140/90 mm Hg are considered normal [11].

Echocardiographic examination: Two-dimensional ultrasound, color Doppler, and tissue Doppler measurements were performed according to the recommendations of the American Society of Echocardiography and the European Association of Cardiovascular Imaging [12,13] using a Siemens ACUSON SC2000 ultrasound system, as previously described [14]. A description of the cardiac malformations detected in 2D was made. Due to the high prevalence of patent foramen ovale in the general population, it was not included as a HD. Briefly, the M-mode method was used to calculate the interventricular septum thickness at end-diastole, left ventricular internal dimension at end-diastole, left ventricular internal dimension at end-systole, posterior wall at diastole, left ventricular mass in grams, right ventricular at end-diastole, left ventricular shortening fraction, and TAPSE. Biplane Simpson’s method was used to calculate LVEF. Mitral inflow Doppler velocities and the peak early (E-wave) and late filling (A-wave) were measured using a pulsed-wave Doppler after placing the sample volume at the leaflets’ tips. The left-ventricle-tissue Doppler velocities, systolic velocity (s’), and diastolic early (e’) and late (a’) lateral mitral annular velocities were calculated after placing the sample volume of the pulsed-wave Doppler at the lateral side of the mitral annulus. The left ventricular diastolic function was evaluated using the E/e’ ratio, lateral e’, tricuspid regurgitation velocity, left atrial volume index, and E/A ratio [13]. The heart disease threshold for LVEF and TAPSE, was calculated based on different guidelines [15,16,17].

Speckle-tracking echocardiography: Images were acquired using the same echocardiography equipment and Velocity Vector Imaging 3.0 software used to obtain the left ventricular GLS measurements, as previously described [14]. Briefly, the cardiac cycle at end-diastole was selected on the echocardiogram, coinciding with the onset of the QRS complex. The four chamber planes of three cardiac cycles were acquired with a comprehensive adjustment of the image quality and a temporal resolution ≥ 60 frames/s. In the case of an inadequate trace, the affected segment was excluded from the analysis, allowing a maximum of two of six segments to be eliminated for the study to be considered valid. Left ventricular GLS (%), strain rate (1/s), and velocity (cm/s) measurements were obtained. Global longitudinal strain and strain rate values are expressed in absolute values. Reference values, according to the different meta-analyses for global longitudinal strain, varied between 15.9% and 22.1%, with a mean of 19.7% and a 95% confidence interval [18,19,20,21,22,23,24].

Statistical analysis: Statistical analyses were performed using the IBM SPSS Statistics^®^ 20.0. software, and graphics were produced with the GraphPad Prism 9 software. Data are presented as median and interquartile range. The comparison between patients with *PACS1*-NDD and the control group was made using the Mann–Whitney U test.

Literature review: We systematically searched the literature in the databases PubMed, Web of Science, and EMBASE from 2012 to 2022. The search strategy included the following key words or terms: *PACS1*, *PACS1* neurodevelopmental disorder, *PACS1*-NDD, Schuurs-Hoeijmakers syndrome, and SHMS. We also manually checked the reference lists from relevant articles and reviews. Trials, case reports, cohort studies, and reviews were included. After full-text reviewing, papers were included if they reported about patients who had a diagnosis of *PACS1*-NDD, according to standard clinical criteria, and carried the pathogenic variant in the *PACS1* gene.

## 3. Results

### 3.1. Baseline Clinical Characteristics and Cardiac Heart Disease Evaluation

Clinical characterizations of the 11 patients are summarized in Table 1. The present cohort comprised five females and six males aged from 2 to 35 years (with a median age of 10 years), with the pathogenic genetic variant c.607C>T, p.(Arg203Trp) in the *PACS1* gene. Most patients presented normal birth parameters with no complications during pregnancy. A single umbilical artery (HP:0001195) was found in two patients (I2 and I4) during prenatal echocardiography. Global developmental delay (HP:0001263) was observed, with language skills being more affected than motor development. Measurements at the last evaluation showed average growth compared to reference standards in the majority of the adolescence and adult patients; however, most of the preschool and childhood patients presented heights of more than two standard deviations below the calculated mean. Heart rate and systolic and diastolic pressure values were within the normal ranges in all individuals evaluated.

**Figure 1:**
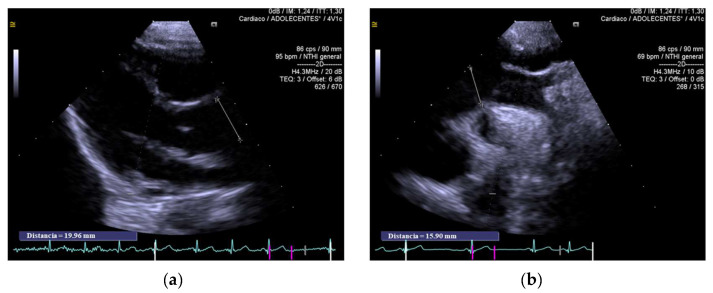
Ascending aortic dilatation in individual I4. (**a**) The parasternal long-axis view of the ascending aorta, showing a mild dilated aorta. Aortic ring, 16.5 mm (z + 3.39); sinus of Valsalva, 22.6 mm (z + 3.06); ascending aorta, 19 mm (z + 2.92). (**b**) Suprasternal view of aortic arch.

**Figure 2:**
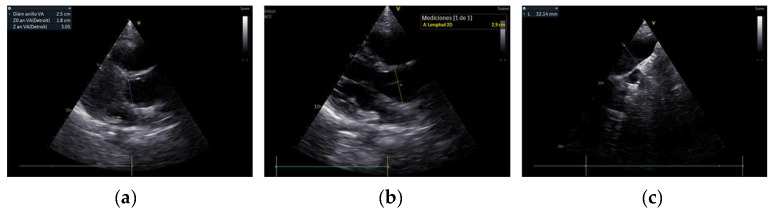
Ascending aortic dilatation in individual I2. (**a**) Parasternal long-axis view of the aortic ring, 25 mm (z + 3.27). (**b**) Sinus of Valsalva, 29 mm (z + 1.5). (**c**) Suprasternal view of aortic arch with ascending aorta, 30 mm (z + 2.94).

Of the 11 individuals examined with echocardiography, 7 presented with HDs (63.6%) (Table 1). Individual I2 presented a muscular ventricular septal defect (HP:0001629) that closed spontaneously during early childhood (at 9 months of age). Patent foramen ovale (HP:0001655) was described in patients I3 and I4 (2 and 4 mm, respectively, both with a left–right shunt). A mild ascending aortic dilatation was detected in three patients: I4 (Figure 1: aortic ring, 16.5 mm (z + 3.39); sinus of Valsalva, 22.6 mm (z + 3.06); ascending aorta, 19 mm (z + 2.92), the aortic valve had three leaflets and no insufficiency), I2 (Figure 2: aortic ring, 25 mm (z + 3.27); sinus of Valsalva, 29 mm (z + 1.50); ascending aorta, 30 mm (z + 2.94); the aortic valve had three leaflets with mild insufficiency), and I10 (aortic ring, 12 mm (z + 82); ascending aorta, 19 mm (z + 3.76); the aortic valve had three leaflets and no insufficiency). Individual I11 had mitral regurgitation secondary to valve prolapse. The only individual who required surgical intervention was I6, owing to an atrial septal defect (HP:0001631) at 10 months of age. Two patients required percutaneous intervention for PDA, at 4 and 18 months of age (I8, I9). None of the patients required pharmacological treatment.

### 3.2. Echocardiographic Findings and Speckle-Tracking-Echocardiographic Parameters

**Figure 3:**
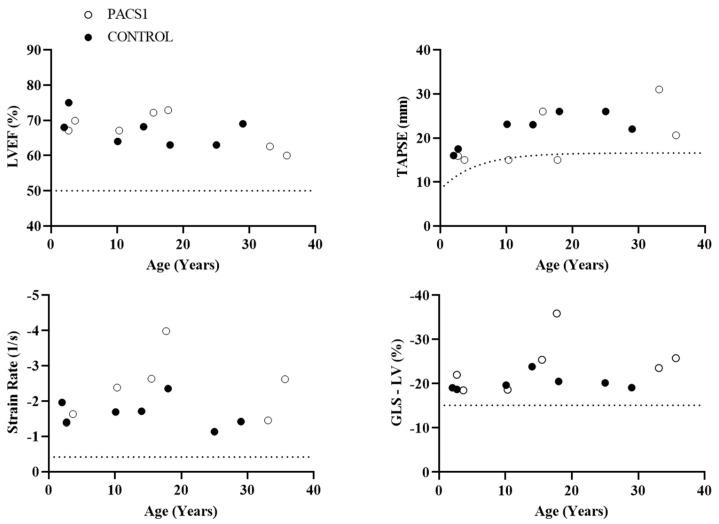
Age evolution of LVEF (%), TAPSE (%), strain rate (1/s), and global longitudinal strain (%) in *PACS1*-NDD (*n* = 7) compared to the control group (*n* = 7). Discontinuous lines show suggested threshold values for heart disease, LVEF [14], TAPSE [16,17], strain rate, and GLS-LV [24].

Heart function assessed by tissue Doppler imaging and two-dimensional speckle tracking were performed in seven patients (I1, I2, I3, I4, I5, I6, and I7) and matched controls. Basic somatometric measurements and echocardiographic parameters of *PACS1*-NDD patients and control subjects are shown in Table S1. Based on echocardiographic parameters, ventricle size, wall thickness, and ventricular ejection fraction appeared to be normal. None of the individuals presented diastolic dysfunction (as mitral pulsed-wave Doppler, lateral tissue Doppler, E/e’ ratio, and E/A ratio were normal). Additionally, the tricuspid regurgitation velocity was <2.8 m/sg and left atrial volume index < 34 mL/m^2^ in all cases. On average, the global strain parameters did not differ significantly between the two groups. Notably, none of the patients showed echocardiographic pathological values, and all of them showed an average global longitudinal strain (GLS) < −15% (Figure 3). Correlation analysis did not reveal a downward trend of strain values (left ventricular GLS), strain rate, left ventricular ejection fraction (LVEF), or tricuspid annular plane systolic excursion (TAPSE) with age in *PACS1*-NDD patients.

### 3.3. Systematic Review

The literature was reviewed to establish an estimate for the prevalence of HD in *PACS1*-NDD (Table S2). With the inclusion of the 11 patients described in this study, HD has been reported in 42% (42/100) of the individuals with *PACS1*-NDD [1,2,3,4,5,8,25,26,27,28,29,30,31,32,33]. Of the 42 patients described with HD, only in 28 the specific defect was described. Septal defects (HP:0001671) are the most common form of HD, accounting for more than 64.28% (18/28) of cases of HD, followed by patent ductus arteriosus (HP:0001643) (28.5%, 8/28). A case of pulmonary artery dilatation was found, but AAD and MVP had not been described in previous studies.

## 4. Discussion

This cohort study evaluating cardiac function by conventional echocardiography and speckle tracking in individuals with *PACS1*-NDD is the largest to date. Moreover, a systematic review focused on determining HD prevalence in *PACS1*-NDD was performed. Our results show that despite the high prevalence of HD (42%), there was no evidence of myocardial dysfunction in our cohort. Echocardiographic values were within the normal range, and myocardial strain parameters did not significantly differ between *PACS1*-NDD patients and individuals in the control group.

*PACS1*-NDD is caused by a recurrent de novo missense variant in the *PACS1* gene (NM_018026.3; c.607C>T, p.(Arg203Trp)) [1]. Although its pathomechanism is still poorly understood, current evidence suggests a dominant negative or a gain-of-function mechanism of the aberrant protein. PACS-1 is a multifunctional connector protein ubiquitously expressed in adult tissues [6]. This protein has been hypothesized to have a role during embryo development because *PACS1* mRNA expression is upregulated, especially in the brain and cerebellum and, to a lower extent, in cardiac muscle tissue [34]. This expression pattern is in line with the main clinical characteristics of *PACS1*-NDD patients: cognitive impairment, dysmorphic facial features, and congenital malformations [4].

Cardiac anomalies are frequently described in *PACS1*-NDD patients. Including the present work, five case studies have analyzed the presence of HD in *PACS1*-NDD [2,3,5,8], with the prevalence ranging from 31.25% to 57.1% across the studies. This variance could be explained by the inclusion or exclusion of patent foramen ovale as a HD. Given that it is present in over 25% of the healthy population [35], excluding this anomaly from the analysis would yield more accurate results. In the current study, the prevalence of HD in *PACS1*-NDD patients was estimated to be 63.6%. More than half of patients with heart defects had septal defects, and another one-third had patent ductus arteriosus. Furthermore, the following heart defects were described in at least one patient: bicuspid aortic valve [27], pulmonary artery dilatation [8], single ventricle [5], coronary artery fistula [33], and, as novelty, three cases of ascending aortic dilatation and one of mitral valve prolapse in our cohort.

CHD is relatively common in syndromic disorders. These abnormalities have been described in syndromes caused both by chromosomal aneuploidies and CNVs [36,37,38], as well as by pathogenic variation of single genes [39]. In fact, in syndromic disorders that are clinically comparable to *PACS1*-NDD, such as CdLS, Kabuki syndrome, and WDR37 syndrome, the prevalence of CHD has been estimated to be 33%, 50%, and 70%, respectively [40,41,42]. In our study, the prevalence of heart disease was high, and although several patients required intervention, they had good subsequent evolution without repercussions on cardiac function to date. Therefore, in contrast to other syndromes in which HD is a major clinical complication [41,43], HDs do not seem to represent an important cause of morbidity and mortality in *PACS1*-NDD or worsen quality of life of the patients. However, in terms of the presence of dilatation of the ascending aorta, one must be cautious and assess the evolution in the future.

To further evaluate subtle cardiac changes in our cohort, in the present study, we determined strain and strain rate parameters derived from two-dimensional speckle-tracking echocardiography. This technique has proven to be useful for the early detection of myocardial dysfunction of the left ventricle in children with different genetic conditions [14,44,45]. Once again, no remarkable pathological findings were observed in any patient or in comparison to the control group. In fact, none of our patients had a GLS > −15.8%, which is a prognostic marker of cardiovascular mortality and morbidity independent of conventional risk factors [46].

One of the main concerns in *PACS1*-NDD evolution is the possibility of a neurodegenerative phenotype. A gradual loss of motor abilities has been observed in some patients [2]. Regarding cardiac function, in our *PACS1*-NDD cohort, we did not observe an age-dependent myocardial dysfunction, a condition that has been described in CdLS [27].

This study has several limitations. The cohort study only included 11 patients. However, *PACS1*-NDD is a very rare disease, and a myocardial strain analysis has never been done before for this syndrome. Another limitation is the huge deviation in the age of the patients, including only two adult individuals. It is possible that subclinical myocardial dysfunction manifestations may become overt with increasing age. Finally, longitudinal studies are needed to evaluate cardiac function in the patients over time.

## 5. Conclusions

Herein, we present 11 patients in a *PACS1*-NDD cohort and review 89 previously reported cases of *PACS1*-NDD. Our data support a high prevalence of HD in *PACS1*-NDD, although the defects are mild and therefore do not represent an important cause of morbidity and mortality or worsening of the quality of life of these patients. AAD and MVP are reported for the first time in this syndrome. Furthermore, a detailed cardiac function evaluation in our cohort using speckle-tracking echocardiography did not reveal evidence of cardiac dysfunction in individuals with *PACS1*-NDD. In spite of this, cardiology evaluation should be included for all individuals with Schuurs-Hoeijmakers syndrome.

## Figures and Tables

**Table 1:** Basic characteristics and cardiac heart diseases of the study group with *PACS1*-NDD (*n* = 11).

Variables	I1	I2	I3	I4	I5	I6	I7	I8	I9	I10	I11
Gender	F	F	F	M	M	M	M	F	F	M	M
Age (y)	33	15	10	2.5	35	17	3.5	6	6	2.8	12.5
Weight (kg)	66.4	52.2 (p37)	35 (p46)	12.6 (p15)	70	52.4 (p9)	10 (*p* < 1, −2.4SD)	17.2 (p11)	16.6 (p8)	9.5 (*p* < 1, −2.5SD)	33 (p8)
Height (cm)	151.5	166 (p70)	140 (p53)	89.5 (p13)	170	175 (p50)	81 (*p* < 1, −4.8SD)	105 (*p* < 1, −2.5SD)	106 (*p* < 1, −2.3SD)	81 (*p* < 1, −2.9SD)	144 (p9)
First words (y)	7	3	2	na	4	5	1.5	6	-	2	3
Age of sitting (m)	8	10	9	20	12	12	11	7	7	14	9
Age of walking (m)	16	16	19	30	24	20	26	36	48	2.5	24
SBP (mmHg)	119	na	119 (p94)	69 (p3)	106	105 (p10)	77 (p23)	na	na	na	na
DBP (mmHg)	69	na	79 (p94)	38 (p31)	78	58 (p20)	52 (p63)	na	na	na	na
HR (bpm)	63	119	77	87	76	93	135	na	na	na	na
HD	no	yes	no	yes	no	yes	no	yes	yes	yes	yes
HD type	-	VSD AAD	-	AAD (PFO)	(PFO)	ASD	-	PDA	PDA	AAD	MVP
Intervention		no		no		yes		yes	yes	no	no

Abbreviations: na—not available, F—female, M—male, SBP—systolic blood pressure, DBP—diastolic blood pressure, HR—heart rate, HD—heart defects (HP:0001627), ASD—atrial septal defect (HP:0001631), VSD—ventricular septal defect (HP:0001629), PFO—patent foramen ovale (HP:0001655), AAD—ascending aortic dilatation (HP:0004942), PDA—patent ductus arteriosus (HP:0001643), MVP—mitral valve prolapse (HP:0001364).

## Data Availability

The data presented in this study are available on request from the corresponding author. The data are not publicly available due to privacy and ethical restrictions.

## Supplementary Materials

The following supporting information can be downloaded at https://www.mdpi.com/article/10.3390/jcm12124052/s1, Table S1: Congenital heart defects described in individuals with *PACS1*-NDD: systematic review of published cases; Table S2: Comparison of echocardiography findings from individuals with *PACS1*-NDD.
