# Supplementary material for: Heart Disease Characterization and Myocardial Strain Analysis in Patients with PACS1 Neurodevelopmental Disorder
Source: J Clin Med. 2023 Jun 14;12(12):4052. doi: 10.3390/jcm12124052 (PMC10299704; doi:10.3390/jcm12124052)
Supplement: Supplementary file 1 [file jcm-12-04052-s001.zip › jcm-2312437-supplementary.pdf]

Table S1: Comparison of echocardiography findings from individuals with *PACS1*-NDD

Variables	PACS1 (n = 7)	Control (n = 7)	p value
Gender (f/m)	3/7	3/7	
Age (y)	15.49 (3.41 - 33.73)	14 (2.70 - 25))	0.7104
Weight (kg)	52.20 (11.95 - 67.30)	45.20 (15 - 67)	0.9709
Height (cm)	151.50 (87.37 - 171.25)	155 (95 - 176)	0.6807
SBP (mmHg)	105.50 (75 - 119)	109 (94 - 118)	0.7115
DBP (mmHg)	63.50 (48.50 - 78.25)	64 (56 - 67)	0.7016
HR (bpm)	87 (72.75 - 103.50)	75 (60 - 89)	0.2745
IVSd (mm)	8.00 (5.67 - 8.70)	7.48 (6.40 - 8.20)	0.7401
LVIDd (mm)	46.00 (31.55 - 48.85)	40.50 (34 - 50.40)	0.9615
LVIDs (mm)	27.20 (20.27 - 30.95)	26.20 (18 - 31.20)	0.8275
PWd (mm)	6.10 (5 - 8)	7.60 (5.44 - 8.50)	0.7815
LVM (g/m^2)	107 (35.47 - 115.25)	81 (47 - 168)	0.9417
RVD (mm)	23 (19.38 - 27.75)	18 (15 - 23)	0.0787
LVEF (%)	67.10 (61.95 - 70.65)	68 (63 - 69)	0.7104
LVFS (%)	36.80 (32.85 - 38.62)	41 (38 - 43)	0.0565
TAPSE (mm)	15.90 (15 - 23.20)	23 (17.5 - 26)	0.2535
E wave (m/s)	0.94 (0.71 - 1.10)	0.90 (0.90 - 1.12)	0.3817
A wave (m/s)	0.51 (0.39 - 0.80)	0.42 (0.40 - 0.51)	0.2471
E/A	1.26 (0.89 - 2.37)	2.20 (1.90 - 2.20)	0.1632
TDI "s" wave (cm/s)	12.60 (11.12 - 15.70)	10 (9 - 10.5)	**0.0233**
TDI "e" wave (cm/s)	14.70 (12.80 - 17.25)	16 (15 - 16)	0.6451
TDI "a" wave (cm/s)	8.40 (4.21 - 8.95)	6 (5.5 - 8)	0.2442
E/e'	5.86 (4.53 - 6.95)	6.58 (5.62 - 7.50)	0.5117
GLS-LV (%)	23.55 (28.32 - 18.62)	9.67 (20.52 - 19.07)	0.3176
Strain rate (1/s)	2.38 (2.96 - 1.43)	1.69 (1.96 - 1.40)	0.2086
Velocity (cm/s)	4.67 (3.03 - 5.30)	3.26 (1.96 - 3.30)	**0.049**

IVSd interventricular septum thickness at end-diastole, *LVIDd* left ventricular internal dimension at end-diastole, *LVIDs* left ventricular internal dimension at end-systole, *PWD* posterior wall at diastole, *LVM* left ventricular mass in grams, *RVD* right ventricular diameter, *LVEF* left ventricular ejection fraction, *LVFS* left ventricular fractional shortening, *TAPSE* tricuspid annular plane systolic excursion, *TDI* lateral mitral tissue Doppler imaging, *GLS-LV* global longitudinal strain for left ventricular function GLS-LV and Strain rate are expressed in absolute values. Values are expressed as median and interquartile range. Groups were compared by Mann–Whitney U test; statistically significant results are highlighted in bold.

Table S2: Congenital heart defects described in individuals with *PACS1*-NDD: systematic review of published cases.

Ref	Type	# Individuals PACS1	Cardiac evaluation	# Individuals with HD	HD description	**ASD**	**VSD**	**PDA**	**PFO**	**BAV**	**PAD**	**SV**	**CAF**	**AAD**	**MVP**
[1]	CR	2	yes	0	-	-	-	-	-	-	-	-	-	-	-
[25]	CR	1	yes	1	yes	1	1	1	0	0	0	0	-	0	-
[2]	CS	19	yes	8	na	-	-	-	-	-	-	-	-	-	-
[8]	CS	8	yes	4	yes	2	3	1	1	0	1	0	-	0	-
[26]	CR	1	na	-	-	-	-	-	-	-	-	-	-	-	-
[27]	CR	1	yes	1	yes	0	0	1	1	1	0	0	-	0	-
[28]	CR	2	yes	1	yes	0	1	0	0	0	0	0	-	0	-
[29]	CR	1	yes	0	-	-	-	-	-	-	-	-	-	-	-
[30]	CR	1	yes	1	na	-	-	-	-	-	-	-	-	-	-
[3]	CS	16	yes	5	na	-	-	-	-	-	-	-	-	-	-
[31]	CR	1	yes	1	yes	0	0	0	1	0	0	0	-	0	-
[5]	CS	35	yes	12	yes	4	4	3	0	0	0	1	-	0	-
[4]	CS	7	na	-	-	-	-	-	-	-	-	-	-	-	-
[32]	CR	1	yes	0	-	-	-	-	-	-	-	-	-	-	-
[33]	CR	1	yes	1	yes	0	0	0	0	0	0	0	1	0	-
Our study	CS	11	yes	7	yes	1	1	2	2	0	0	0	-	3	1
TOTAL		108	100	42	28	8	10	8	5	1	1	1	1	3	1

na no available in the study, *CR* case report, *CS* cases study. Grey shade highlights cases studies. *CHD* congenital heart defects (HP:0001627), *ASD* atrial septal defect (HP:0001631), *VSD* ventricular septal defect (HP:0001629), *PDA* patent ductus arteriosus (HP:0001643), *PFO* patent foramen ovale (HP:0001655), *BAV* bicuspid aortic valve (HP:0001647), *PAD* pulmonary artery dilatation (HP:0004927), *SV* single ventricle (HP:0001750), *CAF* coronary artery fistula (HP:0011641), *AAD* ascending aortic dilatation (HP:0004942), *MVP* mitral valve prolapse (HP:0001364).
